# Plasmodium falciparum and Plasmodium vivax Demonstrate Contrasting Chloroquine Resistance Reversal Phenotypes

**DOI:** 10.1128/AAC.00355-17

**Published:** 2017-07-25

**Authors:** Grennady Wirjanata, Irene Handayuni, Pak Prayoga, Leo Leonardo, Dwi Apriyanti, Leily Trianty, Ruland Wandosa, Basbak Gobay, Enny Kenangalem, Jeanne Rini Poespoprodjo, Rintis Noviyanti, Dennis E. Kyle, Qin Cheng, Ric N. Price, Jutta Marfurt

**Affiliations:** a Global and Tropical Health Division, Menzies School of Health Research, Charles Darwin University, Casuarina, Darwin, Australia; b Papuan Health and Community Development Foundation (PHCDF), Timika, Papua, Indonesia; c Eijkman Institute for Molecular Biology, Jakarta, Indonesia; d District Health Authority, Timika, Papua, Indonesia; e Department of Paediatrics, Faculty of Medicine, Gadjah Mada University, Yogyakarta, Indonesia; f Department of Global Health, University of South Florida, Tampa, Florida, USA; g Australian Army Malaria Institute, Brisbane, Australia; h Clinical Tropical Medicine, Queensland Institute of Medical Research, Brisbane, Australia; i Centre for Tropical Medicine and Global Health, Nuffield Department of Clinical Medicine, University of Oxford, Oxford, United Kingdom

**Keywords:** malaria, Plasmodium falciparum, Plasmodium vivax, drug resistance, chloroquine, chloroquine resistance reversal

## Abstract

High-grade chloroquine (CQ) resistance has emerged in both Plasmodium falciparum and P. vivax. The aim of the present study was to investigate the phenotypic differences of CQ resistance in both of these species and the ability of known CQ resistance reversal agents (CQRRAs) to alter CQ susceptibility. Between April 2015 and April 2016, the potential of verapamil (VP), mibefradil (MF), L703,606 (L7), and primaquine (PQ) to reverse CQ resistance was assessed in 46 P. falciparum and 34 P. vivax clinical isolates in Papua, Indonesia, where CQ resistance is present in both species, using a modified schizont maturation assay. In P. falciparum, CQ 50% inhibitory concentrations (IC_50_s) were reduced when CQ was combined with VP (1.4-fold), MF (1.2-fold), L7 (4.2-fold), or PQ (1.8-fold). The degree of CQ resistance reversal in P. falciparum was highly correlated with CQ susceptibility for all CQRRAs (*R*^2^ = 0.951, 0.852, 0.962, and 0.901 for VP, MF, L7, and PQ, respectively), in line with observations in P. falciparum laboratory strains. In contrast, no reduction in the CQ IC_50_s was observed with any of the CQRRAs in P. vivax, even in those isolates with high chloroquine IC_50_s. The differential effect of CQRRAs in P. falciparum and P. vivax suggests significant differences in CQ kinetics and, potentially, the likely mechanism of CQ resistance between these two species.

## INTRODUCTION

Malaria remains one of the most important infectious diseases in the world, with authorities being notified of 214 million new cases and 438,000 deaths in 2015 (1). Chloroquine (CQ) has been in use for treatment and prophylaxis since the 1940s, but CQ resistance in Plasmodium falciparum was first reported from the Thai-Cambodian border and Colombia within a decade of its deployment, and since then, CQ resistance has spread worldwide (2). Today, WHO recommends that the use of CQ is restricted to non-falciparum malaria in areas with CQ-susceptible infections and the prevention of P. vivax and P. ovale relapses in pregnant and breast-feeding women (3). CQ is still recommended for the treatment of P. vivax malaria in many areas, but its efficacy is threatened by the emergence and spread of CQ-resistant (CQ^r^) P. vivax, with severe public health consequences (4).

Mutations in the *pfcrt* gene encoding the Plasmodium falciparum chloroquine resistance transporter (PfCRT) protein are a major molecular determinant of CQ resistance in P. falciparum (5). It is hypothesized that mutant PfCRT is capable of transporting CQ away from its heme target in the digestive vacuole (DV), thus reducing the level of CQ accumulation in CQ^r^ parasites. The debate as to whether mutant PfCRT acts as a saturable carrier or a voltage-gated channel (reviewed in reference 6) is ongoing. Whereas CQ resistance in P. falciparum has been well studied, little is known about its mechanisms in P. vivax. Candidate markers, such as *pvcrt-o* and *pvmdr1*, have been investigated; however, reports about the association between these molecular determinants and *in vitro* and *in vivo* CQ^r^ phenotypes are conflicting (7–10).

A number of CQ resistance reversal agents (CQRRAs), also known as chemosensitizers, have been identified in P. falciparum *in vitro*. The term “reversal agent” refers to compounds that restore the activity of another drug without having intrinsic activity against the organism (11). The majority of CQRRAs are licensed for use for other medical conditions, including the calcium channel blocker verapamil (VP) (12, 13), the antihistamines chlorpheniramine and cyproheptadine (14–17), the antidepressant desipramine (18, 19), and the neuroleptic compound chlorpromazine (20). Although the mechanisms by which CQRRAs reverse CQ resistance are not fully understood, recent in-depth kinetic studies suggest that VP interacts with multiple binding sites of mutant PfCRT and inhibits PfCRT-mediated CQ transport (21). In addition, the amino acid replacement from lysine to threonine at position 76 of *pfcrt* (K76T) appears to be critical in determining the presence or absence of CQ resistance reversibility by VP, whereas upstream mutations at positions 72 to 75 influence the degree of reversibility (22, 23). Furthermore, reduced reversal activity in the presence of plasma proteins has been reported for desipramine (24) and VP (25). Primaquine (PQ), an 8-aminoquinoline drug currently used to prevent P. vivax relapses and the radical clearance of gametocytes in falciparum malaria, is the only agent with a demonstrable potential to reverse CQ resistance at clinically relevant concentrations (26, 27). More recently, the antidiarrheal agent loperamide, the calcium channel blocker mibefradil (MF), and the neurokinin 1 (NK1) receptor antagonist L703,606 (L7) have been shown to possess even greater potency than VP for reversal of CQ resistance in P. falciparum (28).

Assessment of CQ resistance reversal activity has generally been conducted using P. falciparum laboratory strains or field isolates with well-defined CQ susceptibility phenotypes. This approach is more challenging for P. vivax, since it cannot as yet be sustained in continuous *ex vivo* culture. Hence, the investigation of reversal activity in P. vivax is restricted to the assessment of field isolates, with a single publication reporting a lack of VP reversal activity in a small number of isolates (10).

The current study aimed to investigate the mechanistic differences of CQ resistance-conferring determinants in the two species with a comparative assessment of the activity of VP, MF, L7, and PQ for the reversal of CQ resistance in clinical P. falciparum and P. vivax field isolates.

## RESULTS

### Susceptibility to CQ and CQRRAs.

A total of 80 clinical isolates from patients with single-species infections (46 P. falciparum isolates and 34 P. vivax isolates) were assessed between April 2015 and April 2016. CQ alone and CQ in combination with VP (CQV), MF (CQM), and L7 (CQL) were assessed in all isolates; but the assessment of the combination of CQ plus PQ (CQPQ) was restricted to 26 P. falciparum and 20 P. vivax isolates. CQV data for 14 P. falciparum and 5 P. vivax isolates were excluded from the analysis as the CQ-VP combination in one of the drug plate batches failed to pass the internal drug plate quality control (QC). Adequate growth for harvest (defined as ≥40% schizonts after ≥35 h of incubation) was achieved for 80% (37/46) of P. falciparum isolates and 59% (20/34) of P. vivax isolates. The baseline characteristics of the isolates processed are presented in Table 1.

**TABLE 1:** Baseline characteristics of isolates for which *ex vivo* assays were performed

Baseline characteristic	P. falciparum (*n* = 46)	P. vivax (*n* = 34)
No. (%) of isolates reaching harvest	37 (80)	20 (59)
Median (range) delay from venipuncture to start of culture (min)	150 (65–310)	175 (100–330)
Median (range) duration of assay (h)	43 (35–46)	45 (42–50)
Geometric mean (95% CI^*a*^) parasitemia with asexual-form parasites/μl	21,017 (13,955–31,652)	8,555 (5,656–12,940)
Median (range) initial % parasites at ring stage	100	93 (76–97)
Mean (95% CI) % schizonts at harvest	52 (47–56)	46 (42–49)

a CI, confidence interval.

The 50% inhibitory concentration (IC_50_) of each CQRRA against CQ-resistant (CQ^r^) (K1 and W2) and CQ-sensitive (CQ^s^) (FC27 and 3D7) P. falciparum laboratory strains resulted in intrinsic activities as well as reversal properties similar to those observed in previous studies (Table 2). All CQ resistance reversal agents (CQRRAs) tested possessed weak (micromolar range) intrinsic antimalarial properties across different parasite strains, with the median VP IC_50_s being between 6.5 and 28.5 μM, the median MF IC_50_s being between 1.5 and 8.9 μM, the median L7 IC_50_s being between 5.4 and 12.8 μM, and the median PQ IC_50_s being between 9.6 μM and 14.4 μM. The IC_50_s for these P. falciparum laboratory strains treated with CQ-CQRRAs are presented in Table 2. VP, MF, and L7 showed potent reversal activity against CQ^r^ isolates K1 and W2, reducing the median CQ IC_50_s by approximately 5.6- to 12-fold, 6.3- to 7.6-fold, and 5.1- to 22.7-fold, respectively. In contrast, PQ showed only a modest CQ IC_50_ reduction in K1 and W2 (i.e., 1.6- and 1.5-fold CQ IC_50_ reductions, respectively). CQ IC_50_s for the CQ^s^ strains FC27 and 3D7 were not affected by the addition of any of the CQRRAs tested.

**TABLE 2:** *Ex vivo* drug susceptibility of P. falciparum laboratory strains to CQ and CQRRAs

Drug or drug combination	FC27^*a*^		3D7^*a*^		K1^*b*^		W2^*b*^	
	Median (range) IC_50_ (nM)^*c*^	Fold change^*d*^	Median (range) IC_50_ (nM)^*c*^	Fold change	Median (range) IC_50_ (nM)^*c*^	Fold change	Median (range) IC_50_ (nM)^*c*^	Fold change
VP	27,270 (10,280–34.430)		13,640 (13,630–18,380)		28,530 (13,930–48.570)		6,490 (5,820–6,530)	
MF	8,962 (8,614–9,774)		3,820 (3,760–3,920)		1,544 (1,309–1,699)		2,600 (2,540–2,610)	
L7	12,810 (11,860–24,350)		8,550 (8,430–10,150)		6,466 (5,098–12,372)		5,400 (5,280–5,670)	
PQ^*e*^	NA^*f*^		NA		9,599		14,442	
CQ	9.3 (8.5–10.8)		6.7 (6.6–7.8)		138.9 (116.9–150.2)		174.6 (173.6–181.0)	
CQV (1 μM)	12.8 (11.9–24.3)	0.73	7.3 (6.7–7.6)	0.92	24.6 (20.9–33.1)	5.65	14.6 (14.5–20.2)	11.96
CQM (0.35 μM)	12.1 (11.2–13.9)	0.77	7.8 (6.9–7.9)	0.86	21.9 (14.0–39.3)	6.34	22.9 (22.1–23.7)	7.62
CQL (1.5 μM)	10.8 (10.6–14.9)	0.86	6.9 (6.7–7.6)	0.97	27.2 (14.9–41.9)	5.11	7.7 (7.3–8.2)	22.68
CQPQ (0.5 μM)	7.7 (7.1–7.7)	1.21	11.9 (10.0–20.8)	0.56	86.8 (81.0–96.8)	1.6	120.0 (118.6–138.0)	1.46

a A chloroquine-sensitive laboratory strain.

b A chloroquine-resistant laboratory strain.

c Data are derived from three independent experiments unless indicated otherwise.

d Fold change of the median CQ-CQRRA IC_50_ from the CQ IC_50_.

e Derived from one experiment only.

f NA, not tested in this strain.

**FIG 1:**
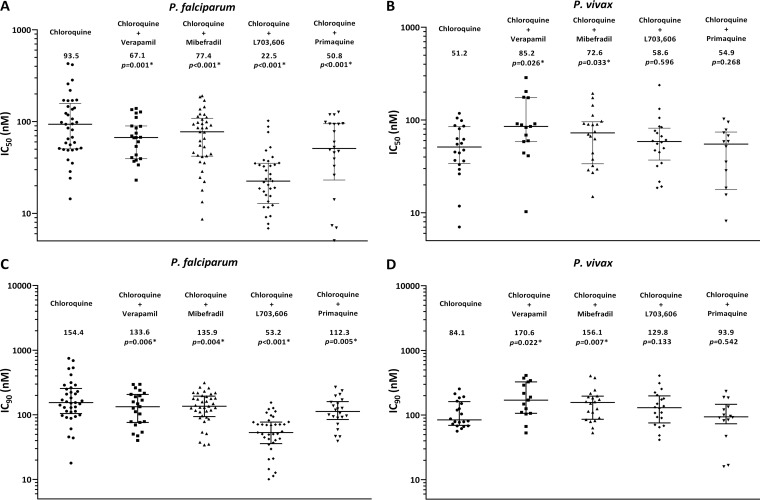
*Ex vivo* drug susceptibility of clinical P. falciparum (left) and P. vivax (right) field isolates. Numbers represent median IC_50_s (in nanomolar) (A and B) and IC_90_s (in nanomolar) (C and D), and error bars represent the interquartile range (IQR). *P* values were derived by the Wilcoxon rank-sum test.

The CQ and CQ-CQRRA IC_50_s and IC_90_s of P. falciparum and P. vivax field isolates are presented in Table 3 and Fig. 1. The median IC_50_ of CQ for 37 P. falciparum isolates was 93.5 nM (range, 14.4 to 427.7 nM), and the median IC_90_ was 154.4 nM (range, 17.9 to 747.2 nM), with 14 isolates (54%) having an IC_90_ of greater than 150 nM. The *ex vivo* susceptibility of P. falciparum to all of the CQRRA combinations was significantly lower than that to CQ alone: 22.5 nM to 77.4 nM for the IC_50_ and 53.2 to 135.9 nM for the IC_90_ (*P* ≤ 0.006 for all comparisons).

**TABLE 3:** *Ex vivo* drug susceptibility of P. falciparum and P. vivax field isolates to CQ and CQRRAs

Drug	P. falciparum				P. vivax			
	IC_50_		IC_90_		IC_50_		IC_90_	
	Median (range) IC_50_ (nM)	*P* value^*a*^	Median (range) IC_50_ (nM)	*P* value	Median (range) IC_50_ (nM)	*P* value	Median (range) IC_50_ (nM)	*P* value
CQ	93.5 (14.4–427.7)		154.4 (17.9–747.2)		51.2 (7.0–118.0)		84.1 (56.3–254.2)	
CQV (1 μM)	67.1 (4.9–139.0)	0.001	133.6 (40.1–296.0)	0.006	85.2 (10.3–286.6)	0.026	170.6 (52.9–409.9)	0.022
CQM (0.35 μM)	77.4 (8.7–190.7)	<0.001	135.9 (34.3–312.8)	0.004	72.7 (14.9–193.8)	0.033	156.1 (53.3–404.2)	0.007
CQL (1.5 μM)	22.5 (2.8–102.0)	<0.001	53.2 (3.5–154.6)	<0.001	58.6 (18.6–237.6)	0.596	129.8 (41.4–407.2)	0.133
CQPQ (0.5 μM)	50.8 (3.1–127.0)	<0.001	112.3 (39.2–268.1)	0.005	54.9 (2.6–102.5)	0.268	93.9 (15.9–232.7	0.542

a *P* values (determined by the Wilcoxon rank-sum test) denote the statistical significance of the difference in median IC_50_s between CQ-CQRRA and CQ.

In the 20 P. vivax isolates, the median *ex vivo* CQ susceptibility was 51.2 nM (range, 7.0 to 118.0 nM) for the IC_50_ and 84.1 nM (range, 56.3 to 254.2 nM) for the IC_90_, with both values being significantly lower than the respective values for the 37 P. falciparum isolates (*P* = 0.005 and *P* = 0.009, respectively) and with 6 isolates (30%) having an IC_90_ of greater than 150 nM. In contrast to the effects of the CQRRAs in P. falciparum, in P. vivax isolates there was no significant difference in the IC_50_s with compounds L7 and PQ and a small increase in the median IC_50_s with VP and MF (85.2 nM [*P* = 0.026] and 72.7 nM [*P* = 0.033], respectively; Table 3).

### Association between degree of reversal activity and CQ susceptibility.

**FIG 2:**
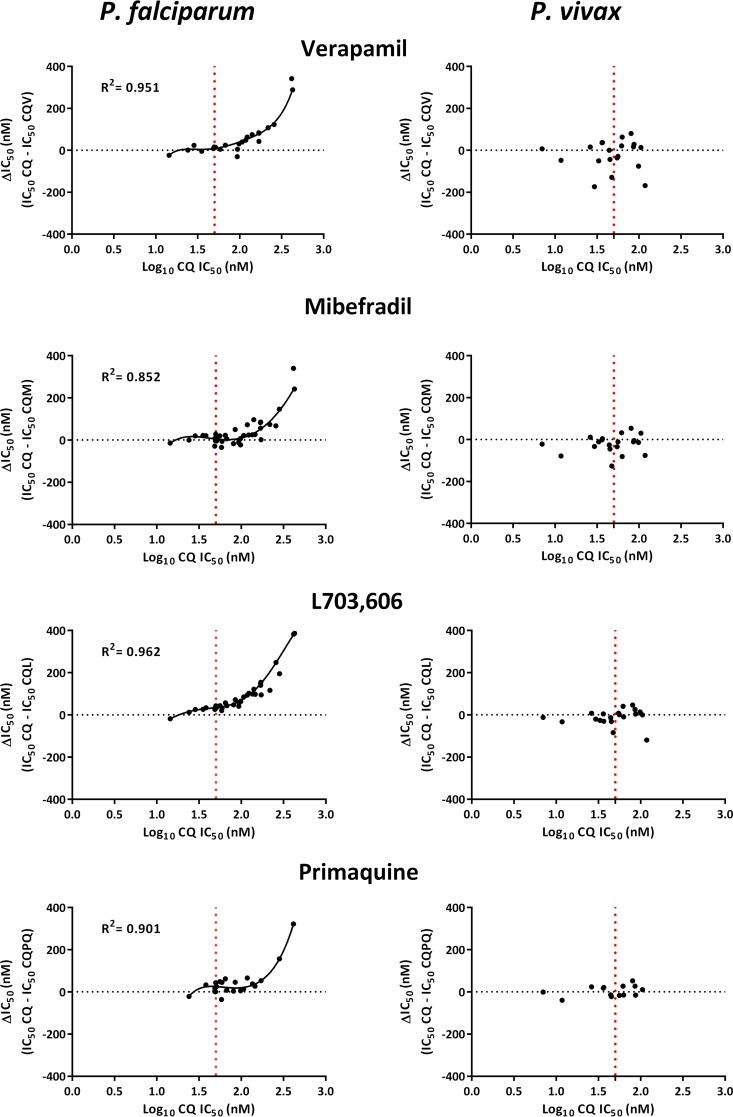
Polynomial regression analysis between ΔIC_50_ (the CQ IC_50_ minus the CQ-CQRRA IC_50_) and the CQ IC_50_. Data were analyzed using a third-order polynomial regression model. The vertical dotted lines denote the inflection point of the CQ IC_50_ in P. falciparum and the corresponding concentration in P. vivax. In P. vivax, the data failed to fit a polynomial regression model of any order. *R*^2^ values represent the coefficients of determination.

Overall, the CQ IC_50_ for the P. falciparum isolates was correlated with the change in the IC_50_ (ΔIC_50_) compared to the IC_50_s with CQV (*R*^2^ = 0.951), CQM (*R*^2^ = 0.852), CQL (*R*^2^ = 0.962), and CQPQ (R^2^ = 0.901) (*P* < 0.001 for all comparisons). The data were best fitted to a third-order polynomial regression model with inflection points at CQ IC_50_s of 44 nM for CQV and CQM, 42 nM for CQL, and 67 nM for CQPQ. In contrast, there was no correlation between the CQ IC_50_ for the P. vivax isolates and the ΔIC_50_ for any of the tested CQRRAs; this was also apparent in the 10 (50%) isolates with CQ IC_50_s above the inflection points (42 to 67 nM) observed in P. falciparum (Fig. 2).

## DISCUSSION

Our comparative study confirms the ability of four known CQRRAs to reverse CQ resistance in P. falciparum laboratory strains, and this was also apparent in the CQ^r^ P. falciparum clinical isolates (Fig. 1). The reduction in CQ susceptibility with MF and VP was more modest in the clinical isolates (1.2- and 1.4-fold, respectively) than the laboratory strains (10- and 6-fold, respectively). Whereas PQ showed similar reversal activity in both laboratory strains and clinical isolates (∼1.5-fold), L7 had the most potent reversal activity in clinical isolates (almost 4-fold). In contrast, none of these CQRRAs significantly increased CQ susceptibility in the P. vivax isolates under similar experimental conditions.

In P. falciparum, the reversal of CQ resistance is associated with increased levels of accumulation of CQ in the parasite's digestive vacuole (DV) and a reduction of CQ IC_50_s, and this is most apparent in CQ^r^ strains (13, 29). The potency of CQRRAs in reversing CQ resistance is influenced by the genetic background of the parasites, with pfCRT variants playing a critical role (23, 30). Although pfCRT is a key determinant of both CQ resistance and the ability of chemosensitizers to reverse this resistance, both mechanisms are likely to be modulated by other factors, including variability at other genetic loci (31, 32). All of the P. falciparum isolates in this study had a CQ^r^ *pfcrt* isoform (i.e., CVIET and SVMNT; data not shown), and this may, in part, explain the differences in the potential to reverse CQ resistance observed between the laboratory strains and the clinical isolates. Furthermore, whereas laboratory strains presented CQ susceptibility phenotypes at the extreme ends of the spectrum, the clinical isolates were much more diverse and showed a range of IC_50_s. Clinical infections can be caused by a complex mixture of parasites, with up to 10 parasite clones being found per infection (33, 34). Our clinical isolates were specifically selected for *ex vivo* phenotyping, all came from patients with moderate to high levels of parasitemia, and a majority of the parasites were at the ring stage. Approximately 20% of P. falciparum infections and 35% of P. vivax infections were polyclonal (unpublished data), and these isolates are more likely to consist of a mixture of CQ^r^ and CQ^s^ parasite clones than monoclonal isolates or laboratory strains are. This may have accounted for the greater variability in the potential to reverse CQ resistance that we observed in this field study.

The relationship between CQ susceptibility and the potential of verapamil, mibefradil, L703,606, and primaquine to reverse CQ resistance was explored using polynomial regression. Whereas the degree of CQ resistance reversibility was a function of CQ susceptibility in P. falciparum, no apparent relationship between CQ resistance reversal potential and baseline CQ susceptibility was observed in P. vivax, even in isolates with IC_50_s greater than the inflection point observed in P. falciparum.

Clinical studies have demonstrated a high degree of CQ resistance in Papua, Indonesia, and Papua New Guinea (35, 36). In a study in Timika, Papua, Indonesia, conducted in 2005, 65% of patients had a recrudescent infection within 28 days of treatment with chloroquine monotherapy, with early failure occurring in 15% of patients (37). In view of this high level of CQ resistance, antimalarial treatment policy was changed to dihydroartemisinin-piperaquine in 2006, and it has not been ethical to repeat a clinical trial of CQ therapy since that time. Thus, continued surveillance for CQ resistance has relied on monitoring of clinical isolates for *ex vivo* drug susceptibility. The definition of *ex vivo* CQ resistance using a universal threshold for the IC_50_ has significant limitations, particularly when comparing different drug susceptibility assays, such as the [^3^H]hypoxanthine incorporation and schizont maturation assays. We have shown previously that the *ex vivo* CQ susceptibility in Papua (where there are known P. vivax isolates with high-grade CQ resistance *in vivo*) and Thailand (where CQ is clinically effective against P. vivax), determined using the same assay methodology, differed accordingly, with the isolates in Papua having significantly higher IC_50_s than those in Thailand (10).

Our ongoing *ex vivo* surveillance of CQ susceptibility suggests that while IC_50_s have fallen slightly and are indeed lower than those documented for P. falciparum (Table 3), a significant proportion of the P. vivax clinical isolates remain CQ resistant. Clinical and pharmacology studies suggest that the growth of parasites in whole-blood concentrations of greater than 100 ng/ml is indicative of CQ resistance (38). If it is assumed that the MIC corresponds to an *ex vivo* IC_90_ of approximately 150 nM, then at least 6 of the 20 (30%) P. vivax isolates assessed in the current study would be categorized as highly CQ resistant. There was no significant difference in either the IC_50_ or the IC_90_ in these isolates when any of the CQRRAs was added to CQ *ex vivo*.

The mechanisms of CQ action and resistance in P. vivax and, hence, the potential to reverse CQ resistance in this species remain unknown. Sà et al. have demonstrated that the 3D7 P. falciparum strain transformed with the *pfcrt* orthologue *pvcrt-o* showed 2.2-fold increased CQ IC_50_s and this effect could be reversed with VP (9). More recently, the activities of two “reversed chloroquine” (RCQ) compounds (PL69 and PL106) were assessed against P. falciparum and P. vivax isolates collected from the same area where the isolates for the current study were collected, and the two species showed different profiles of susceptibility to the two compounds (39). Whereas the IC_50_s of both compounds were significantly lower than the IC_50_ of CQ alone in P. falciparum, only PL69 showed some, albeit weak, reversal activity in P. vivax, suggesting that the imipramine-like moiety of PL69 may have an effect on CQ activity in P. vivax. Further investigations on CQ kinetics will be needed to elucidate how P. vivax evades CQ toxicity.

In conclusion, all CQRRAs tested in this study demonstrated reversal activity in CQ^r^ P. falciparum isolates, with the recently identified pharmacophore L703,606 being the most potent. There was no corresponding reduction in chloroquine susceptibility in P. vivax. The results of the study suggest differences in CQ kinetics between the two Plasmodium species, with the lack of reversal activity in P. vivax supporting a growing body of work suggesting that the molecular determinants of CQ resistance in these two species may be different (40, 41).

## MATERIALS AND METHODS

### Study area and field sample collection.

The study was conducted in Timika, Papua Province, Indonesia, between April 2015 and April 2016. Patients presenting to the Rumah Sakit Mitra Masyarakat (RSMM) Hospital were enrolled into the study if they were diagnosed with a microscopically confirmed P. falciparum or P. vivax monospecies infection with a level of parasitemia of between 2,000 and 80,000 parasites/μl. Plasmodium isolates were processed only if at least 60% of asexual forms were at the ring stage. Patients were excluded if they had been treated with CQ in the last 30 days or any other antimalarial treatments in the last 2 weeks or had a hemoglobin concentration below 5 g/dl. After written informed consent was obtained, 5 ml of venous blood was collected and processed immediately. Host white blood cells were removed using commercially available Plasmodipur filters (Europroxima B.V., Arnhem, The Netherlands) according to the manufacturer's instructions, and the packed infected red blood cells (iRBC) were used for the *ex vivo* drug susceptibility assays.

### Evaluation of intrinsic antimalarial activities of CQRRAs.

Verapamil hydrochloride (VP; Sigma-Aldrich, Australia), mibefradil hydrochloride (MF; Tocris Bioscience, UK), L703,606 (L7; Sigma-Aldrich, Australia), and primaquine (PQ; World Wide Antimalarial Resistance Network [WWARN] QA/QC Reference Material Programme [42]) were selected as CQ resistance reversal agents (CQRRAs) in this study. The intrinsic antimalarial activities of the recently reported MF and L7 had hitherto been tested only in the CQ^r^ strain K1 (28). Therefore, prior to the experiments in field isolates, the intrinsic antimalarial properties of these reversal agents, in addition to those of VP and PQ, were assessed in CQ^r^ (K1 and W2) and CQ^s^ (3D7 and FC27) P. falciparum laboratory strains (BEI Resources, ATCC, Manassas, VA, USA).

### Drug compounds and drug plate preparation.

Each drug plate (96 wells) contained 11 serial concentrations (2-fold dilutions) in duplicate. Predosed drug plates were made by adding 25 μl of drug dilutions to each well, followed by lyophilization and storage at 4°C. The maximum concentration of CQ (provided by the WWARN QA/QC Reference Material Programme [42]) was 2,993 nM. In order to minimize the intrinsic antimalarial activities of the CQRRAs but retain their reversal activities, the concentrations of CQRRAs in experiments with multiple laboratory strains and P. falciparum and P. vivax field isolates were set as follows. For VP, the concentration added to each serial CQ dilution was derived from previous experiments performed by Martin et al. (13) and Martiney et al. (43) (i.e., 1,000 nM), and for PQ, it was derived from previous findings by Bray and colleagues (i.e., 500 nM) (26). The fixed concentrations of MF (0.355 μM) and L7 (1.5 μM) added to each serial CQ dilution were based on the lowest IC_50_s observed across the multiple parasite strains tested (Table 2).

### *Ex vivo* drug susceptibility assay.

The drug susceptibilities of the Plasmodium isolates were measured using a modified schizont maturation assay as described previously (39, 44). Drug susceptibility profiles were presented as inhibition of parasite growth from the ring stage to the schizont stage. Two hundred microliters of a 2% hematocrit blood medium mixture (BMM), consisting of RPMI 1640 medium supplemented with 10% human serum (P. falciparum) or McCoy's 5A medium supplemented with 20% human serum (P. vivax), was added to each well of the predosed 96-well drug plates. The parasites were cultured in a candle jar at 37.0°C for 35 to 50 h. Incubation was stopped when more than 40% of the ring-stage parasites had reached the mature schizont stage in the drug-free control well. The plates were harvested by preparing thick blood films from each well of the plates. Thick blood films were stained with 5% Giemsa solution for 25 min and examined microscopically. Differential counts of 200 asexual-form parasites in the test slides were classified into ring, trophozoite, and mature schizont stages. To reduce parasite classification error and to standardize parasite identification between readers, only schizonts with at least 5 or more well-defined chromatin dots were classified as mature schizonts at harvest. Free merozoites and gametocytes were not included in the count. To determine the effect of each antimalarial drug, the schizont count at each drug concentration was normalized with that of the corresponding drug-free control well.

### Data analysis.

Drug response data were analyzed using the *in vitro* analysis and reporting tool (IVART), a free and automated online platform to produce IC_50_ estimates by applying nonlinear regression analysis (45). Final data analysis was performed using STATA (version 14.1; Stata Corp., College Station, TX) and GraphPad Prism (version 6; GraphPad Software, Inc.) software. The Wilcoxon signed-rank test, polynomial regression analysis, and Spearman rank correlation were used for nonparametric comparisons.

### Ethical approval.

Ethical approval for this project was obtained from the Human Research Ethics Committee of the NT Department of Health & Families and the Menzies School of Health Research, Darwin, Australia (HREC 2010-1396), and the Eijkman Institute Research Ethics Commission, Jakarta, Indonesia (EIREC 47 and EIREC 67).
